# Successful therapeutic strategy for a patient with obese end-stage kidney disease by simultaneous laparoscopic sleeve gastrectomy and implantation of a buried peritoneal dialysis catheter: A case report

**DOI:** 10.3389/fmed.2022.926652

**Published:** 2022-09-23

**Authors:** Tomohisa Yamashita, Tatsuya Sato, Kazuyuki Yamamoto, Atsuko Abiko, Keitaro Nishizawa, Masahiro Matsuda, Yuma Ebihara, Takeshi Maehana, Toshiaki Tanaka, Toshiyuki Yano, Hironori Kobayashi

**Affiliations:** ^1^Department of Cardiovascular, Renal and Metabolic Medicine, Sapporo Medical University School of Medicine, Sapporo, Japan; ^2^Department of Nephrology, Japanese Red Cross Asahikawa Hospital, Asahikawa, Japan; ^3^Department of Cellular Physiology and Signal Transduction, Sapporo Medical University School of Medicine, Sapporo, Japan; ^4^Department of Surgery, Japanese Red Cross Asahikawa Hospital, Asahikawa, Japan; ^5^Department of Diabetic and Endocrinologic Medicine, Japanese Red Cross Asahikawa Hospital, Asahikawa, Japan; ^6^Department of Gastroenterological Surgery II, Faculty of Medicine, Hokkaido University, Sapporo, Japan; ^7^Department of Urology, Sapporo Medical University School of Medicine, Sapporo, Japan

**Keywords:** end-stage kidney disease, obesity, laparoscopic sleeve gastrectomy, a buried catheter for peritoneal dialysis, kidney transplantation, personalized therapeutic approach

## Abstract

For morbidly obese patients with end-stage kidney disease (ESKD), there are often difficulties in accessing, implementing, and maintaining kidney replacement therapy (KRT). Although recent weight-loss surgery has the potential to solve these problems, its therapeutic strategy and appropriate perioperative management for morbidly obese patients with ESKD have not been established. Here, we describe the case history of a 47-year-old man diagnosed with ESKD due to obesity-related glomerulopathy with an uncorrected estimated glomerular filtration rate (eGFR) of 16.1 ml/min. He hoped for kidney transplantation but was not eligible due to his high body mass index (BMI) (36.9 kg/m^2^). Therefore, a combination strategy for both attaining weight loss and preparing for KRT was needed. We performed modified laparoscopic sleeve gastrectomy (LSG) combined with a buried catheter for peritoneal dialysis (PD), which resulted in reduction of multiple surgical invasions while simultaneously preparing for PD. After these operations, his body mass dropped to below 30.0 kg/m^2^, making him a candidate for kidney transplantation, while maintaining PD. Finally, he was able to have kidney transplantation with success. Collectively, in this case, our novel therapeutic approach was able to avoid multiple surgeries, to assist catheter insertion by laparoscopy, and to provide optimal KRT for an obese patient with ESKD. Simultaneous LSG and implantation of a buried PD catheter may be a promising strategy for morbidly obese patients with ESKD.

## Introduction

The increasing prevalence of obesity has been a leading public health problem worldwide and has posed formidable challenges in accessing, delivering, and maintaining optimal kidney replacement therapy (KRT) in patients with end-stage chronic kidney disease (ESKD) (1, 2). Kidney transplant recipients for patients with morbid obesity are at a higher risk of postoperative complications, including delayed graft function, wound infection, and rejection (3). Thus, a recent clinical practice guideline Kidney Disease: Improving Global Outcomes (KDIGO) has recommended that kidney transplant candidates with obesity should be offered weight loss interventions prior to transplantation (4). However, longer and more frequent hemodialysis (HD) or peritoneal dialysis (PD) is required to achieve adequate clearance until weight loss is achieved, frequently leading to problems with vascular access and catheter insertion for maintaining HD or PD in obese patients with ESKD (1, 5, 6). Despite these problems in KRT for morbidly obese patients with ESKD, safe and effective therapeutic strategies have not been established.

Recent pre-transplant bariatric surgery may be one promising option for weight loss interventions prior to transplantation (7, 8). A recent retrospective cohort study revealed that the intervention of bariatric surgery in patients on HD or PD was associated with an increased rate of kidney transplants performed but also lower all-cause mortality at 5 years compared with usual care (9). However, in obese patients with advanced chronic kidney disease or ESKD not undergoing HD or PD, surgery-related kidney damage and perioperative complications associated with vascular access and catheter insertion are critical problems despite early surgical intervention being paradoxically required.

The stepwise initiation of PD using a Moncrief and Popovich buried catheter (SMAP) is a technique for implanting a PD catheter in a standard fashion, except that the external segment is buried subcutaneously, and for exteriorizing the catheter when the initiation of PD is required (10). We hypothesized that simultaneous execution of PD catheter insertion with laparoscopic sleeve gastrectomy (LSG) may be ideal for morbidly obese patients with ESKD for the following three reasons. First, PD can be initiated without additional procedures in cases in which kidney function worsens after bariatric surgery. Second, laparoscopy allows surgeons to monitor the positioning of the catheter tip within the peritoneal cavity and assists PD-related surgical procedures including rectus sheath tunneling and lower abdominal suture sling (5, 6). Third, peritoneal adhesions can be prevented by avoiding multiple surgeries.

In this report, we describe a case of successful kidney transplantation *via* PD induction in an obese patient with ESKD using our novel therapeutic strategy by simultaneous LSG followed by SMAP.

## Case report

A 47-year-old Japanese man was referred to Japanese Red Cross Asahikawa Hospital because his estimated glomerular filtration rate uncorrected for body surface area for Japanese individuals (uncorrected eGFRcreat) decreased to 16.1 ml/min. He was 178 cm tall, weighed 117 kg, and had a body mass index (BMI) of 36.9 kg/m^2^, which is categorized as morbidly obese. Although he had been diagnosed with diabetes, hypertension and dyslipidemia, he was being appropriately treated with anti-diabetic agents, anti-hypertensive agents, and statins. He had edema in his lower limbs. His laboratory data at the first visit are shown in Table 1. Laboratory data revealed a serum creatinine level of 4.64 mg/dL, potassium level of 5.1 mmol/L and corrected calcium level of 7.4 mg/dL. Urinalysis showed a protein-to-creatinine ratio (PCR) of 7.4 g/gCr without occult blood. Endocrinological tests revealed no evidence of secondary obesity. No diabetic or hypertensive retinopathy was observed by an eye examination. These findings led to the clinical diagnosis of obesity-related glomerulopathy, though the diagnosis was not proven by kidney biopsy.

**TABLE 1:** Laboratory data at the first visit.

Parameter	Level	Parameter	Level	Normal range
*Complete blood count*		*Endocrinological test*		
White blood cell, x10^3^/μL	9.9	Thyroid-stimulating hormone, μIU/mL	3.63	861–1,747
Neutrophils, %	73.7	Free thyroxine 4, ng/dL	1.16	93–393
Hemoglobin, g/dL	12.8	Cortisol, μIU/mL	16.8	33–183
Platelets, × 10^3^/μL	205	Adrenocorticotropic hormone, pg/mL	44.7	73.0–138.0
*Serum biochemistry*		Plasma renin activity, ng/mL/h	2.7	11.0–31.0
Total protein, g/dL	6.1	Aldosterone, ng/dL	2.0	30.0–50.0
Albumin, g/dL	3.4	Growth hormone, ng/mL	0.27	<3.5
Urea nitrogen, mg/dL	49.9	Fasting insulin, μIU/mL	25.5	<3.5
Creatinine, mg/dL	4.64	C-peptide immunoreactivity, ng/mL	14.41	<2.0
Sodium, mmol/L	142			
Potassium, mmol/L	5.1	Urinalysis		
Chloride, mmol/L	109	Protein-to-creatinine ratio, g/gCr	7.43	
Calcium, mg/dL	6.8	Red blood cells, per high power field	0–1	
Phosphate, mg/dL	4.5	White blood cells, per high power field	1–4	
Fasting blood glucose, mg/dL	98			
Hemoglobin A1 c,%	5.9			

**FIGURE 1:**
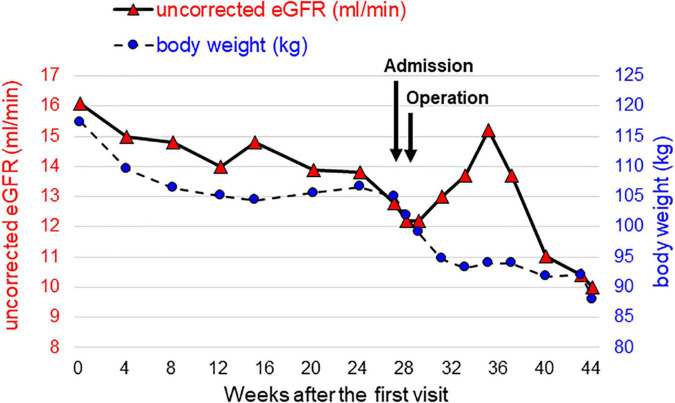
Time courses of kidney function and body weight from the time of the initial visit to the introduction of peritoneal dialysis. Uncorrected eGFR, which reflects kidney function, is represented by red triangles on the left axis, and body weight is represented by blue circles on the right axis.

Although he hoped for a kidney transplant as KRT, he was not eligible due to his high BMI. Therefore, we needed to assist his weight loss safely while maintaining his kidney function well. We considered that pre-transplant LSG was the optimal way for successful kidney transplant. The time courses of kidney function and body weight after the first visit are shown in Figure 1. A preoperative low-calorie diet (LCD) for 5 months decreased his body weight by 12 kg. However, his uncorrected eGFRcreat decreased to 12.8 ml/min, suggesting that other KRT is required for maintaining his kidney function before kidney transplantation. Since he selected PD as 1st-line KRT, we planned modified LSG combined with a buried catheter for PD simultaneously. Two weeks before the operation, he was admitted to our institute to control his total daily energy intake and to restrict the total amount of potassium to less than 1,500 mg per day. At the time of admission, his body weight and urinary PCR were 105 kg and 5.2 g/gCr, respectively. He received a formula-based liquid meal replacement LCD substituting one meal per day (1,520 kcal-containing 53 g protein) in the first week. After confirmation that serum potassium was in the optimal range by taking sodium zirconium cyclosilicate (SZC) at 11.5 g per day, the frequency of liquid meal replacement was increased to two meals per day (950 kcal-containing 50 g protein) in the second week. His body weight dropped to 99 kg, and his blood pressure was normalized, leading to discontinuation of administration of antihypertensive drugs.

Following the preoperative medical treatment, bariatric surgery for obese ESKD by simultaneous LSG followed by SMAP was performed.

**FIGURE 2:**
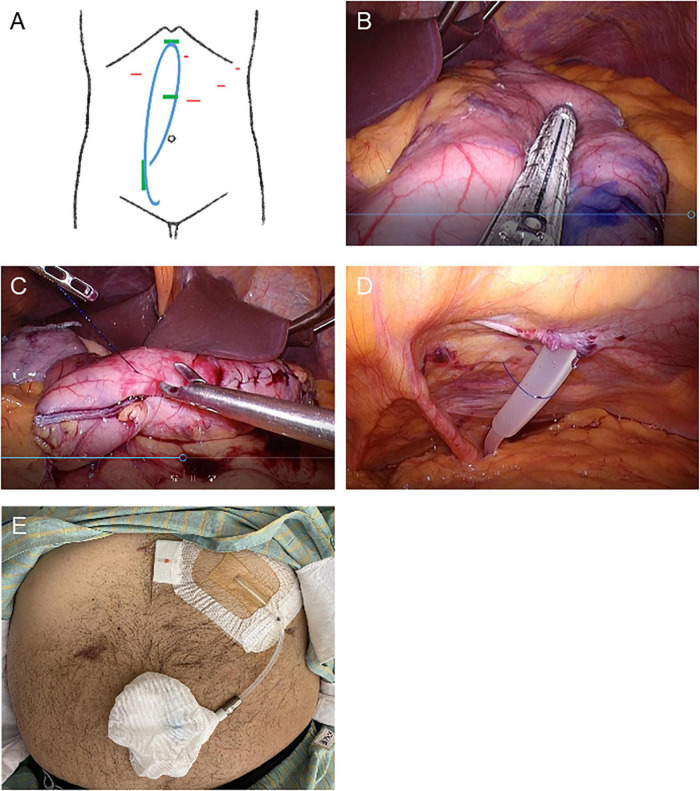
Schematic images and intraoperative photographs of simultaneous laparoscopic sleeve gastrectomy and implantation of a buried peritoneal dialysis catheter. **(A)** Schematic designs of simultaneous laparoscopic sleeve gastrectomy and implantation of a buried peritoneal dialysis catheter. Red: location of trocar insertion. Blue: buried PD catheter. Green: PD catheter insertion and implantation site. **(B–D)** Intraoperative photographs. **(B)** Longitudinal sleeve gastrectomy from the antrum to the proximal His angle. **(C)** The stapler line sutured. **(D)** PD catheter sutured laparoscopically to the lower abdominal wall. **(E)** Photograph of the PD catheter exteriorized to the upper abdomen.

While each method used for LSG (11) and SMAP (10) mostly complied with those previously described, the detailed procedure for the simultaneous LSG followed by SMAP in the present case is as described below. The patient was placed in the supine reverse Trendelenburg position with his legs apart under general anesthesia. Four trocars were placed to avoid the planned PD catheter implantation site, and a 15 mm port was subsequently extended to extract the specimen (Figure 2A). The gastrocolic ligament along the greater curvature of the stomach was opened using a vessel sealing system (LigaSure System; Covidien, Mansfield, MA, USA) and exposed to the esophagogastric junction. A 37 Fr bougie was then inserted into the pylorus, and an endoscopic linear stapler was used, starting with a black cartridge at the antrum and proceeding proximally to the angle of His using purple cartridges (The Endo GIA Reinforced Reload with Tri-Staple Technology; Covidien) (Figure 2B). The stapler line was oversewn with a running 3–0 absorbable suture to prevent bleeding, leakage, and adhesions (Figure 2C). Intraoperative endoscopy was used to confirm the absence of leakage. Then we inserted and buried a 65 cm non-coiled Swan-Neck PD catheter with three cuffs [JL-2 (A)S3, Hayashidera Medinol, Kanazawa, Japan] into the peritoneal cavity. The intra-abdominal segment of the PD catheter was led to the Douglas pouch under laparoscopic guidance without using a stylet and sutured to the lower abdominal wall (Figure 2D). A tunnel was created from the skin incision to the epigastric fossa to imbed the subcutaneous segment. Subsequently, another tunnel was created to lead the catheter to the first skin incision. A drainage tube was placed along the suture line of the stomach. Finally, the PD catheter was filled with heparinized saline (100 IU/10 mL), plugged, and successfully buried. A gastrografin swallow test was performed on the 5th postoperative day and we confirmed that there was no gastric leak.

One week after surgery, his body weight and BMI had decreased to 95 kg and 30.0 kg/m^2^, respectively. Finally, he was able to be listed as a kidney transplant candidate. His urinary PCR was decreased to 3.5 g/gCr 1 month after surgery. His uncorrected eGFRcreat was temporarily increased to 14.8 ml/min but was then decreased to 10.0 ml/min 3 months after surgery (Figure 1). Therefore, the PD catheter was exteriorized to the upper abdomen (Figure 2E) and a full volume exchange was able to be initiated without any trouble. The patient had been on PD while awaiting kidney transplantation. He was able to have kidney transplantation in Sapporo Medical University Hospital 14 months after the first visit. During this clinical course, he had no recurrent weight gain or complications.

## Discussion

We found three possible valuable advantages of this strategy for an obese patient with ESKD by simultaneous LSG and implantation of a buried PD catheter through this case history. First, it enables safe and assured introduction of PD in parallel with LSG. Second, the number of surgeries can be reduced, resulting in less invasive procedures for morbidly obese patients with ESKD, who are usually at very high risk for operation-associated complications. Third, the patient’s weight can be reduced at the time of PD induction, allowing sufficient PD efficiency to be achieved from the beginning. Therefore, our novel strategy (Bariatric surgery for Obese end-stage REnAl disease by simultaneous LSG followed by SMAP, which we named “BOREASS technique”) can provide patient-friendly KRT. Furthermore, we showed two benefits in perioperative medical management for obese patients with ESKD through the present case history. First, we decreased total daily energy intake using a formula diet, which resulted in decreased progression of kidney function and avoidance of using a very LCD that can lead to distress and risks of muscle wasting for the patient (12). Second, serum potassium levels were successfully maintained within the normal range by using SZC without any side effects including gastrointestinal adverse events (13).

Removal of solute and fluid by PD is generally thought to be less efficient than that by HD, which contributes to a shorter duration to transfer to HD from PD in obese patients than in lean patients (14). Thus, our strategy may also contribute to extension of the PD duration, if the patient wishes for PD, by attaining safe and effective weight loss with maintenance of good nutrition status. Furthermore, obesity makes creating vascular access more challenging and complex, possibly by physical compression and impeding its maturation (1). Thus, our strategy using the BOREASS technique can facilitate vascular access creation and serve as a bridge to combination therapy with HD and PD. We believe that this technique is also useful for ESKD patients not only for kidney transplantation but also for PD or combination therapy.

It has been reported that bariatric surgery has renoprotective potentials by multiple mechanisms including the metabolic and anti-hypertensive effects (15). Thus, the PD catheter can be preserved without usage for years when kidney function is reserved after surgery. Gupta et al. reported that a 20-year-old buried catheter was successfully used for PD initiation (16). Therefore, we consider that our novel BOREASS technique can be applied for patients with not only ESKD but also preserved-stage chronic kidney disease who require preservation of kidney function.

A major advantage of performing simultaneous LSG and implantation of a buried PD catheter is that the surgeon can place the intra-abdominal segment of the PD catheter into the Douglas pouch under laparoscopic guidance. In addition, in the present case, abdominal sling suture was also performed to prevent PD catheter migration. Although rectus tunneling, another method to prevent PD catheter dislodgement (6), was not performed in the present case, no trouble with the position of the PD catheter occurred during the period of 14 months until the patient underwent kidney transplantation. We acknowledge that ESKD patients with obesity may have a large omentum, leading to catheter entrapment, and thus the option of omentectomy or omentopexy may prevent future PD catheter obstruction. In the present case, omentopexy was not performed because low surgical invasiveness and short surgical time were prioritized. Although no catheter obstruction occurred in the present case, further studies are needed to determine whether omentectomy or omentopexy in simultaneous LSG and PD catheter insertion can result in beneficial outcomes or not. Regarding the PD catheter exit site, we chose the upper abdominal exit site to avoid body hair on the whole abdomen, as the patient desired. Indeed, an upper abdominal exit has the advantage that it is easier for the patient to see and care for the exit site (6). However, we consider that an upper abdominal exit is not necessarily needed in our BOREASS technique. The exit site should be chosen with consideration of the comfort and preference of the patient.

The most common and life-threatening complication of LSG is gastric leak (17). Thus, to prevent gastric leak, we routinely perform intraoperative endoscopy and a gastrografin swallow test on the 4th or 5th postoperative day as in the present case. In addition, fever and tachycardia, the most important clinical signs with gastric leak, should be monitored carefully. If gastric leak occurs, we perform nasogastric tube decompression and, if necessary, endoscopically guided endoluminal vacuum therapy (18). Other procedures such as endoscopic double-pigtail catheter internal drainage can also be applied (19). We do not believe that simultaneous LSG and PD catheter insertion should change these management strategies, but future studies are needed to determine the optimal way for preventing complications.

As an important limitation of our report, the rate of adverse events caused by our BOREASS technique has not been assessed to date because we present only one obese Japanese patient with ESKD. Recent prospective data collected between 2017 and 2018 in the United States revealed that the absolute event rate of death within 30 postoperative days by bariatric surgery is below 0.5% in patients with stage 4 or 5 chronic kidney disease (20). To establish the usefulness and safety of our novel strategy, prospective cohort studies with a sufficient sample size are needed in the future.

In summary, our strategy by simultaneous LSG and implantation of a buried PD catheter may be an optimal therapy for obese patients with advanced chronic kidney disease and ESKD. Further studies are needed to establish the usefulness of our novel approach.

## Data availability statement

The raw data supporting the conclusions of this article will be made available by the authors, without undue reservation.

## Ethics statement

Ethical review and approval were not required because this is not a clinical study but a case report in accordance with the local legislation and institutional requirements. The patients/participants provided their written informed consent to participate in this study. Written informed consent was obtained from the individual(s) for the publication of any potentially identifiable images or data included in this article.

## Author contributions

TMY conceived the original idea. TMY and TS drafted the manuscript and prepared for table/figures. KY and YE were responsible for LSG. HK was responsible for PD catheter insertion/exteriorization. AA was responsible for nutrition support. TMY, KN, MM, and HK maintained PD. TM and TT were responsible for kidney transplantation. TSY and HK supervised the clinical courses. All authors provided important intellectual content during manuscript drafting and contributed to the finalization of the manuscript.
